# Opioid Prescribing Practices and Quality Improvements Within an Outpatient Orthopedic Practice

**DOI:** 10.7759/cureus.14332

**Published:** 2021-04-06

**Authors:** Cyrus M Nouraee, Rachel M Uzlik, Gary E Wyard, Rebecca Stone McGaver, Dean M Ericksen, Ryan A Moon, M. Russell Giveans

**Affiliations:** 1 Orthopedic Surgery Research, Revo Health, Golden Valley, USA; 2 Quality, Revo Health, Golden Valley, USA; 3 Orthopedic Surgery, Twin Cities Orthopedics, Golden Valley, USA; 4 Statistics, Twin Cities Orthopedics, Golden Valley, USA

**Keywords:** opioid prescribing, outpatient surgery, ambulatory surgery center

## Abstract

Background

During the last several decades, the opioid epidemic throughout the United States has been recognized as an increasing problem. The aim of this study was to identify and implement processes throughout a single private orthopedic practice and managed ambulatory surgery centers to reduce opioid prescription pill quantity and strength, while also reducing consumption.

Methodology

A baseline assessment along with the development of four separate phases was implemented. Data collection included type, dosage, and quantity of opioids prescribed after elective outpatient procedures as well as patient interviews/surveys within two weeks after surgery. Quality improvement implementation included: (a) presentations on opioid prescribing at an individual physician level, (b) internal prescription guidelines, (c) required Prescription Monitoring Program registration, and (d) patient narcotic education pamphlets after surgery. The average opioids prescribed and consumed were compared between different time points.

Results

Analysis revealed a highly statistically significant decrease in both pills and morphine equivalent units (MEUs) prescribed (p < 0.001, p < 0.001) between the baseline assessment and four subsequent phases, as well as consumed (p < 0.001, p < 0.001) between phases one through four. Even though patients were consuming less pills and MEUs than they were prescribed on average across all phases, overall pain levels increased between phases one through four (p < 0.001), and overall satisfaction of pain control decreased between phases two through four (p < 0.001).

Conclusions

Over a 24-month time frame, a single private orthopedic practice set a goal of reducing prescribing habits and with successful implementation of various measures, a significant reduction in opioids prescribed and consumed was accomplished. Interestingly, pain level and satisfaction of pain control worsened even though patients were continuing to be prescribed more opioids on average than they were consuming. Therefore, it may be normal to see these results when attempting to set the expectation for some level of pain and reduced consumption of opioid medications post-operatively. Overall, these results can be useful to healthcare administrators and surgeons looking for ways to combat the opioid epidemic.

## Introduction

The United States (US) is in the grip of a tragic opioid epidemic. Between the years of 1999 and 2017, there have been around 400,000 deaths from opioid overdoses [1]. In 2017, there were over 138 opioid overdose deaths each day [2]. Approximately 21 to 29% of patients who are prescribed opioids for pain misuse them [3].

Among the opioid prescriptions dispensed throughout all US retail pharmacies in 2009, orthopedic surgeons accounted for an estimated 7.7% or 6.1 million opioid prescriptions [4]. They were the third highest prescribers of opioids behind primary care physicians and dentists [4]. The prescribing of opioid pain medication for post-operative pain is one of the most common events that leads to opioid abuse and eventually addiction [5,6]. Therefore, it is paramount that orthopedic surgeons decrease their prescribing practices of opioids (dosing and strength) in an effort to reduce these unintended consequences.

Accordingly, at Twin Cities Orthopedics (TCO), one of the largest (115 physicians) private orthopedic practices in the US [7], the surgeons and leadership focused on leading a change in the crisis. The surgeons had two critical questions: (a) how variable are prescribing practices among providers (surgeons and physician assistants) in the practice and (b) can reduction of prescribing quantities and consumption quantities occur with the implementation of internal quality improvement initiatives?

At the start of the project, no interventions were imposed. The aim of this project was to identify and implement processes throughout a single private orthopedic practice and managed ambulatory surgery centers (ASC) that encouraged and reduced opioid prescription pill quantity, strength, and dose, as well as opioid consumption.

## Materials and methods

A quality improvement project (retrospective chart review as well as prospective patient interviews/surveys) was approved through an institutional review board. The project included four separate phases after baseline assessment and initially began in the summer of 2017 and concluded in the summer of 2019. The project was initially hypothesized by a small group of physicians questioning how pain was managed for their patients with a desire to understand the quantity and specific type of opioid being prescribed. Following development, a unanimous approval was given by ASC boards and the TCO board to evaluate current internal processes throughout the entire organization. Quality improvement interventions were implemented between phases, and the timing of phases and interventions were prospectively selected. Patient collection was focused exclusively on ASC patients who underwent an elective outpatient orthopedic surgical procedure performed by a TCO surgeon within a Revo Health-managed ASC. Excluded ASC cases were procedures involving Tenex, epidural steroid injections, and outpatient total joint replacements (knee, hip, shoulder, ankle, and disc).

Phases

Baseline assessment of prescribing included a retrospective chart review of surgical cases done across three ASCs between January 2016 and June 2016. Patients were randomly selected using Microsoft Excel randomization calculation. Data collection involved the type, dosage, and quantity of opioids prescribed after the surgical cases. Upon evaluation of these data points, the data were compared to the Centers for Disease Control (CDC) 2016 guidelines [8], and organizational phases and goals were set forth to reduce the quantity of pills being prescribed within the organization.

Phase one, initiated after the baseline, involved all consecutive surgeries throughout three Revo Health-managed ASCs during a two-week period starting at the end of July 2017. Prescription data of all surgical cases during this time period were reviewed. Patient consumption data were obtained by calling patients; initially 14 days ± three days post-operatively. Patients verbally consented and were asked about the utilization of their prescribed opioids. Phone calls were conducted for two business weeks and patients were called back up to three times if they did not answer. Information collected included the opioid type, dispensed dosage, and quantity of opioids prescribed after each surgical case, along with questions asked to the patient regarding their current pain level (using the Numerical Rating Scale) after the procedure [9], how many pills they had taken since the surgery, and how many refills they had received.

Phase two involved all patients having surgeries at the same three ASCs during a two-week period in December 2017. Information collected was the same as it was in phase one with the addition of asking patients their satisfaction of pain control after surgery (Numerical Rating Scale) [10]. Following this phase of data collection, TCO implemented formal internal prescription guidelines.

Phase three included all surgeries at one of the five ASCs (including the three ASCs previously viewed in phases one and two) in May, June, and July 2018. Data collection otherwise matched the parameters of phase two criteria.

Phase four included all patients who responded to an electronic post-operative medication survey following their elective outpatient orthopedic surgeries at the same five ASCs. Responses were collected for a one-year time period (August 23, 2018 to August 23, 2019). Responses collected matched those of phases two and three even though responses were collected via survey instead of patient interviews. Data analysis continued to monitor if there was a statistically significant decrease in average prescribing and consumption quantities for all procedures, surgeons, and facilities.

Interventions

**Figure 1:**
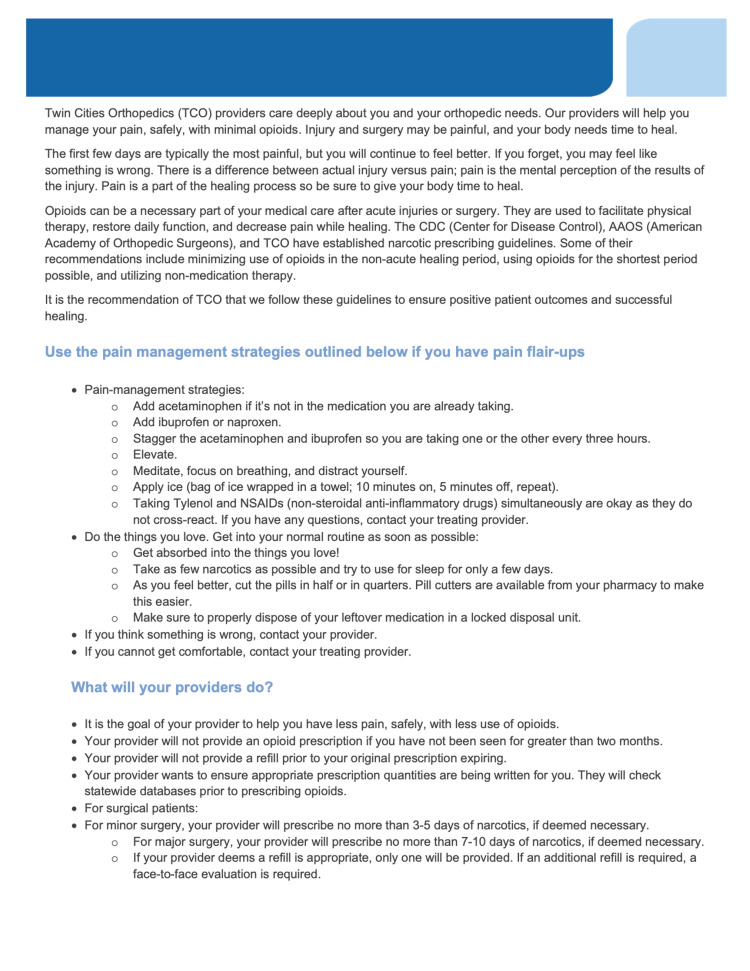
Patient opioid education pamphlet.

Quality improvement initiatives included numerous interventions to help combat overprescribing and overconsumption of opioids post-surgically. First, all surgeons within the practice were presented the collected data at the procedure level after baseline and each phase of data collection. The presented data included individual surgeons’ average prescriptions (in morphine equivalent units [MEUs] and pills) benchmarked alongside the average for the practice for every procedure. Prescription guidelines for the individual procedures were also benchmarked alongside the other averages following development after phase two. Presentations occurred at all physician meetings and data were presented by the Chief Medical Officer and senior leadership. This transparent ranking report was key and well accepted by all physicians. Second, internal prescription guidelines (Table 1) were established following phase two from both patient consumption data and CDC guidelines for acute pain which suggested that MEU dosages should be no greater than 50 MEUs per day, and that clinicians should prescribe opioids at the lowest effective dose and for no longer than the expected duration of pain [11]. These guidelines were distributed to all providers and implemented in all clinics and ASCs throughout the entire organization after phase two. Additionally, between phases two and three, all providers were required to have Prescription Monitoring Program (PMP) registration. Third, between phases two and three, patient opioid education pamphlets were also developed and given to every patient for acute pain following outpatient surgical cases (Figure 1). The pamphlets informed the patient of the organization’s new recommendations for prescribing opioids as well as pain management strategies.

**Table 1:** Opioid guideline chart by drug and maximum MEUs for the ten most common orthopedic procedures. MEUs: morphine equivalence units; ORIF: open reduction internal fixation; ACL: anterior cruciate ligament; RCR: rotator cuff repair

	Norco 5/325 mg	Norco 10/325 mg	Percocet 5/325 mg	Dilaudid 2 mg	MS Contin 15 mg	Oxycontin 10 mg	Oxycodone 5 mg	Tylenol #3	Tramadol 50 mg
ORIF distal radius and ulna (Max 150 MEUs)	10-30	5-15	6-20	6-20	2-6	3-9	6-18	11-33	10-30
Carpal tunnel release (0 MEUs)	0	0	0	0	0	0	0	0	0
Laminectomy (Max 150 MEUs)	10-30	5-15	6-20	6-20	2-6	3-9	6-18	11-33	10-30
Ankle scope with debridement (Max 114 MEUs)	22	11	15	14	5	7	15	25	22
Hip scope debridement (Max 150 MEUs)	10-30	5-15	6-20	6-20	2-6	3-9	6-18	11-33	22-33
Anterior cruciate ligament repair (Max 168 MEUs)	22-33	11-16	15-22	14-21	5-7	7-11	15-22	25-37	22-33
Knee scope with medial or lateral meniscus repair (Max 100 MEUs)	20	10	13	12	4	7	13	22	20
Knee scope with meniscectomy (Max 100 MEUs)	20	10	13	12	4	7	13	22	20
Shoulder scope with decompression (Max 114 MEUs)	22	11	15	144	5	7	15	25	22
Shoulder scope with RCR (Max 225 MEUs)	45	15-25	20-33	18-31	6-11	10-25	20-33	22-44	30-50

Statistical analysis

One-way analysis of variance and Tukey post-hoc tests, where applicable, were used to determine significant differences in prescribing habits between baseline and all four phases. Statistical analyses were performed with SPSS version 24 (IBM Corp., Armonk, NY, USA), and significance was set at p < 0.05.

## Results

Throughout baseline and phases one through four, the average ages of patients were clinically similar (range, 46.7-51.3 across phases), and females represented 50% or more of the population. Baseline assessment of 876 patients demonstrated 50% (438) females with an average age of 48.8 years. Of the 630 patients reviewed in phase one, there were 357 (56.7%) females with an average age of 49.0 years. Phase two revealed 549 surgical patients with 282 (51.4%) females and an average age of 46.7 years. Phase three included 1,473 total charts being reviewed and 56.3% were females (829) with an average age of 49.7 years. Phase four noted females as 59.2% (2236) and an average age of 51.3 years.

Overall, there was a statistically significant decrease in both pills prescribed (p < 0.001) and MEUs prescribed (p < 0.001). There was a statistically significant decrease in pills prescribed between baseline and each of phases one through four (p < 0.001) (Table 2). Additionally, there was a significant decrease in pills prescribed between phases two and three (p < 0.001) and phases two and four (p < 0.001) (Table 2). A statistically significant decrease in MEUs prescribed was seen between baseline and each of the phases one through four (p < 0.001) (Table 2). There was a significant decrease in MEUs prescribed between phase two and phase three (p < 0.001) and phase two and phase four (p < 0.001) (Table 2).

**Table 2:** The total cohort of patient prescriptions (pills and MEUs). MEUs: morphine equivalence units *indicates a significant decrease from baseline; ^indicates a significant decrease from phase two

	Baseline	Phase one	Phase two	Phase three	Phase four
Total number of charts reviewed	876	630	549	1,473	3,777
Number of pills prescribed (mean)	46.08	37.23*	33.41*	28.73*^	27.62*^
Number of pills prescribed (range)	0-150	0-130	0-120	0-120	0-100
MEUs prescribed (mean)	324.53	261.98*	222.83*	190.16*^	184.68*^
MEUs prescribed (range)	0-1,350	0-1,500	0-975	0-1,080	0-900

There was also an overall decrease in both pills consumed (p < 0.001) and MEUs consumed (p < 0.001). There was a statistically significant decrease in pills consumed between phases two and four (p = 0.022) but not between phases two and three (p = 0.092) (Table 3). There was not a statistically significant difference seen in MEUs consumed between phases two and three (p = 0.186) or phases two and four (p = 0.053) (Table 3).

**Table 3:** Summary of patient responses with pills and MEUs consumed across all phases. MEUs: morphine equivalence units ^indicates a significant decrease from phase two

	Phase one	Phase two	Phase three	Phase four
Total number of responses	181	169	346	3,777
Number of pills consumed (mean)	21.18	17.56	14.25	14.14^
Number of pills consumed (range)	0-150	0-95	0-108	0-100
MEUs consumed (mean)	150.75	118.2	97.3	95.91
MEUs consumed (range)	0-1,320	0-822.5	0-907.5	0-750

Reported pain levels for phases one through four were 1.73, 1.15, 1.52, and 2.49, respectively. There was a significant improvement in pain levels between phases one and two (p = 0.008); however, this worsened from phase two to phase four. Between phases two and three, the trend worsened (p = 0.054). Between phases three and four and overall between phases one and four, pain levels worsened (p < 0.001, p < 0.001). Satisfaction for phases two through four was 9.34, 8.88, and 8.09, respectively. Overall, satisfaction scores significantly worsened. There was a trend towards worsening between phases two and three (p = 0.054), between phases three and four (p < 0.001), and between phases two and four (p < 0.001).

Between each phase, providers moved towards lower strength opioids and reduced the quantity of pills being prescribed. Between baseline and phase one, there was a 19% reduction in both the average number of pills and MEUs being prescribed. The four-month time frame between phases one and two resulted in a reduction in prescribing pill quantities by 10% and reduced average MEUs being prescribed by 15%. There was a reduction between phases two and three and between phases three and four in both pills (14% and 4%, respectively) and MEUs prescribed (15% and 3%, respectively). The overall change from baseline to phase four resulted in a 40% reduction in pills prescribed and a 43% reduction in MEUs prescribed.

## Discussion

There has been much reporting on opioid prescribing, use, and abuse throughout the decades [4,12-14]. Theils et al. studied over 7,600 patients undergoing elective surgery for the 25 most commonly performed procedures at three hospitals (seven out of 25 of these procedures were orthopedic procedures) [15]. They reported that there are large variations in prescribing practices at the procedure and provider level that cannot be accounted for by patient-specific factors [15]. Yet, interventions to combat both the over-prescribing and over-consumption of opioids have not been extensively studied outside of the implementation of formal prescribing guidelines for individual procedures.

A 2018 study by Wyles et al. showed that institutional prescribing guidelines developed by inter-departmental healthcare professionals for common orthopedic procedures decreased opioid prescribing [16]. These results, in addition to the results of a handful of other studies looking at the implementation of formal prescription guidelines for orthopedic surgery, show that the implementation of prescribing guidelines are effective in reducing post-operative prescriptions of opioids when looking at a procedure-specific level [17]. It is important to note that the results of these studies are smaller-scale and procedure-specific. To our knowledge, no study has looked at the implementation of formal prescription guidelines across an organization, which is more interesting for healthcare administration looking to make overall systemic changes to their organizations. This current study found similar results to the smaller-scale, procedure-specific studies as the prescribing of opioids significantly decreased. Moreover, there was a significant decrease in both pills and MEUs prescribed between phases two and three and phases two and four (Table 2), where the additional interventions were the implementation of formal prescription guidelines (Table 1) and required PMP registration. Both of these measures likely contributed to the decrease in the prescription of pills and MEUs seen. The results of this study add to the current knowledge by showing that implementation of formal guidelines for the most common procedures in a practice can indeed help to decrease opioid prescriptions. Moreover, PMP registrations can help to decrease opioid prescriptions as well. These are important for healthcare organization leadership to consider, especially when trying to reach a goal of decreasing opioid prescriptions.

Additionally, this current study utilized presentations to physicians. The average pills and MEUs prescribed were significantly decreased when comparing baseline to all four phases (Table 2). Therefore, these presentations likely had some effect in decreasing prescribing by making physicians more aware of their own prescribing and the potential waste that was being prescribed. In the context of the opioid epidemic, this could have helped to change prescribing behaviors. This measure has important implications and should also be considered by healthcare organization leadership when trying to reach a goal of decreasing opioid prescriptions.

No study to our knowledge has shown the effect of patient education pamphlets on post-operative orthopedic surgery opioid consumption across a practice (compared to consumption pre-implementation). While Dwyer et al. implemented patient education regarding safe and effective opioid use, they did not compare consumption to a baseline assessment so no conclusions could be drawn [18]. This current study shows that the implementation of patient opioid education pamphlets (Figure 1) can in fact help to decrease patient consumption of opioids as evidenced by the overall significant decrease in consumption of pills and MEUs across the study. In addition, after implementation of the pamphlets, there was a trend towards decreasing the consumption of pills between phase two and phase three and a significant decrease in the consumption of pills between phase two and phase four (Table 3). There was a trend towards decreasing the MEUs consumed between phases two and three and phases two and four, but this was not significant (Table 3). While there was not a significant decrease shown between phases two and three for pills and MEUs consumed and between phases two and four for MEUs consumed, these results can be due to the difficulties of implementing the patient education pamphlets. Educating all ASC staff members on the newly available resource and training staff to regularly distribute it to patients took time. Further studies are needed to completely understand the full benefits of patient narcotic education handouts, but this study does show some positive benefit with respect to patient consumption. Thus, healthcare administrators looking to decrease patient opioid consumption may consider utilizing patient opioid education pamphlets (Figure 1).

It is interesting to note that while prescribing practices and consumption patterns of opioid pain medications decreased across this project, satisfaction of pain control also decreased and pain level increased. There was an improvement in pain levels between phases one and two, but this then trended towards worsening (between phases two and three) and then worsened (between phases three and four). There was also a trend towards worsening satisfaction of pain control between phases two and three and worsened thereafter. These trends in pain levels and satisfaction of pain control could be attributed to the expectation set forth that post-operative pain is normal and the fact that patients were consuming less opioid pain medications. Additionally, external factors such as the media’s focus on addressing the opioid epidemic may have played some role in patients consuming less opioids and having more pain [19]. On average, patients were being prescribed adequate amounts of opioids as the amount of opioids prescribed was still in excess of that consumed in phases three and four. Thus, some degree of increased pain or decreased satisfaction of pain control may be normal when trying to combat post-operative opioid prescribing. These results can provide helpful insight to other practices or organizations when enacting changes in their opioid prescribing practices.

Limitations in our project included provider-level communication, design of the project, and the media’s role. At the provider level, physician assistants (PAs) were sometimes prescribing post-operative medications. Communicating guidelines to PAs following phase two proved to be challenging at times. Although the surgeons had been informed about these measures, PAs had not. In regards to the design, consistency was important to have throughout. Patient interviews, though a script was followed, may have yielded variations; considerations were given on the organization’s staff to not lead the patient to answer the questions in a certain way. Also, phases one through three utilized patient phone interviews while phase four utilized an online questionnaire allowing patients to complete their responses from home without staff support or oversight. Lastly, the project occurred during the time when media coverage of the opioid epidemic increased drastically [19]. These influences could have played a role in why prescribing practices and consumption behaviors both decreased outside of the mechanisms of change listed in the project. Additionally, recent state regulations regarding inappropriate prescribing of opioids have led to providers possibly prescribing less opioids out of concern for disciplinary action [20]. This additional fear could be an outside factor influencing the decrease in prescribing of opioids seen in the current project.

## Conclusions

In conclusion, overprescribing habits of opioids will remain a problem until physicians and organizations implement alterations within their systems. During a 24-month time frame, a single private orthopedic practice set a goal to reduce prescribing and consumption of opioids and had success with implementation of internal objectives: (a) presentations on opioid prescribing at an individual physician level, (b) internal prescription guidelines, (c) required PMP registration, and (d) patient narcotic education pamphlets after surgery. The successes seen in this single private orthopedic practice can provide insight into implementation that can be useful to other organizations looking to combat the opioid epidemic.
